# Dr. Alonzo Boice

**Published:** 1898-04

**Authors:** 


### DR. ALONZO BOICE.

Died at Kingston, N. Y., after a lingering illness, Dr. Alonzo Boice, of Philadelphia.

Dr. Boice was born March 12, 1847, in the town of Canajoharie, Montgomery County, N. Y.

He commenced the study of dentistry at the early age of fifteen, with Dr. Frisselle, of Kingston, N. Y., and subsequently graduated at the Philadelphia Dental College in 1869, and was for a time demonstrator of mechanical dentistry in that institution.

He was a member of the American Dental Association, Pennsylvania State Dental Society, the Odontographic Society, the Odontological Society, the Historical Society of Pennsylvania, Philadelphia County Dental Society, etc.

The activity of Dr. Boice in dental work gave him more than a local reputation, and his personal acquaintance throughout the country was large.

His work was peculiar, in that he seemed to delight most in controversy, and hence appeared to take special gratification in advancing dentistry, not by the slower process of scientific research, but by force of law. He was, therefore, a prominent worker wherever laws were to be enforced. While this was his peculiar and most prominent characteristic, he was not slow to appreciate the value of the more scientific side of his profession, which was made evident by his close attention to convention work.

The writer of this frequently came into collision with Dr. Boice from an honest difference in views, but at no time was there ever a loss of respect, for whatever may have been thought of the methods used by him, he was always regarded as a man true to his convictions, and in proportion as an individual follows these is he worthy of honor. The energetic honest fighter against abuses, as he understands them, is a loss to any community, and it is felt that the void made by the death of Dr. Boice will not easily be filled in Philadelphia.

